# Screening and brief intervention for alcohol use disorder risk in three middle-income countries

**DOI:** 10.1186/s12889-022-14358-4

**Published:** 2022-10-26

**Authors:** Mallie J. Paschall, Christopher L. Ringwalt, Deborah A. Fisher, Joel W. Grube, Tom Achoki, Ted R. Miller

**Affiliations:** 1 PIRE Programs NF, Pacific Institute for Research and Evaluation, 2030 Addiston St., Suite 410, 94704 Berkeley, CA United States; 2 PIRE Programs NF, Pacific Institute for Research and Evaluation, 101 Conner Drive, Suite 200, 27514 Chapel Hill, NC United States; 3 PIRE Programs NF, Pacific Institute for Research and Evaluation, 4061 Powder Mill Road, Suite 350, 20705 Beltsville, MD United States; 4 Curtin University School of Public Health, 6845 Perth, WA Australia; 5 AB InBev Foundation, 1440 G Street NW, 20005 Washington, DC United States

**Keywords:** Alcohol use screening, Brief intervention, Alcohol use disorder, Middle-income countries

## Abstract

**Background:**

This study examined the prevalence of screening and brief intervention (SBI) for alcohol use disorder (AUD) risk in samples of adult drinkers in three middle-income countries (Brazil, China, South Africa), and the extent to which meeting criteria for AUD risk was associated with SBI.

**Methods:**

Cross-sectional survey data were collected from adult samples in two cities in each country in 2018. Survey measures included past-year alcohol use, the CAGE assessment for AUD risk, talking to a health care professional in the past year, alcohol use screening by a health care professional, receiving advice about drinking from a health care professional, and sociodemographic characteristics. The prevalence of SBI was determined for past-year drinkers in each country and for drinkers who had talked to a health care professional. Logistic regression analyses were conducted to examine whether meeting criteria for AUD risk was associated with SBI when adjusting for sociodemographic characteristics.

**Results:**

Among drinkers at risk for AUD, alcohol use screening rates ranged from 6.7% in South Africa to 14.3% in Brazil, and brief intervention rates ranged from 4.6% in South Africa to 8.2% in China. SBI rates were higher among drinkers who talked to a health care professional in the past year. In regression analyses, AUD risk was positively associated with SBI in China and South Africa, and with brief intervention in Brazil.

**Conclusion:**

Although the prevalence of SBI among drinkers at risk for AUD in Brazil, China, and South Africa appears to be low, it is encouraging that these drinkers were more likely to receive SBI.

**Supplementary information:**

The online version contains supplementary material available at 10.1186/s12889-022-14358-4.

## Background

Harmful use of alcohol is responsible for 3 million deaths globally each year and constitutes the leading cause of premature mortality and disability among individuals aged 15 to 49. It particularly affects disadvantaged and vulnerable populations [1]. Since the 1980s, the World Health Organization has endorsed Screening and Brief Intervention (SBI) as a key prevention strategy targeting harmful drinking [2]. SBI has also been recommended by both the Centers for Disease Control and Prevention (CDC) [3] and the U.S Substance Abuse and Mental Health Services Administration [4]. SBI is administered by health care practitioners to identify and intervene with patients at risk for hazardous or harmful drinking. Hazardous drinkers are those *at risk* of alcohol-related harm, whereas harmful drinking encompasses those who have experienced harm due to their drinking but are not manifesting symptoms of dependence [5].

SBI is based on the assumption that individuals with an elevated risk for hazardous or harmful drinking may be unaware of the effects of alcohol on their own and others’ physical or mental health and may respond positively to brief counseling by a trusted medical provider who offers guidance on how to reduce their consumption [6]. The screening tool for SBI typically uses one of two instruments: the Alcohol Use Disorders Identification Test (AUDIT) or the Cut Down, Annoyed, Guilty, Eye-Opener (CAGE) [4]. Practitioners are encouraged to engage patients who score above a specified threshold on either of these brief screeners in a brief 5-to 10-minute intervention. It is recommended that the intervention should include a warning that the patients’ alcohol consumption may have a negative effect on their health, offer practical suggestions as to how they may reduce their drinking, encourage them to reduce their alcohol intake, increase their confidence that they can make any desired changes, and develop a plan to reduce their drinking [5].

Evaluations of SBI demonstrated positive results, including in middle-income settings, and suggested that the cost effectiveness of the intervention may be substantial [7–11]. A Cochrane systematic review of 34 studies conducted in 2018 found that patients receiving brief interventions delivered within the context of general practice or emergency care settings, relative to controls, reduced hazardous and harmful drinking up to 12 months later [12]. However, two recent reviews found only modest effects of brief interventions on alcohol use, with diminishing effects after 6 and 12 months [13, 14]. Noting the decline in effect sizes reported over time, the author of a recent commentary concluded that SBI alone should not be expected to affect population health related to alcohol use, particularly in the face of what he called “conceptually crude” advice delivered hurriedly and within the context of a wider “alcogenic” environment conducive to alcohol use and misuse [15]. Despite growing concerns about SBI’s benefits, there have been no calls to abandon it by institutional sponsors and it remains popular among health care practitioners. Indeed, the need for strategies like SBI is only likely to increase over time, even if their population-level effects may be difficult to detect [5, 15].

Largely unaddressed by previous studies, which have generally focused on SBI’s *effects*, are a range of questions related to SBI’s *prevalence* at the population level. As Rosário and colleagues [16] have suggested, health practitioners’ failure to screen their patients for potentially hazardous and harmful drinking wastes an opportunity to identify at-risk drinkers and invite them to consider modifying behaviors that may be deleterious to their health. Several studies have examined this missed opportunity from practitioners’ perspectives. For example, Wilson and colleagues [17] reported that 40% of sampled general practitioners in England reported that they asked patients about their alcohol use “almost all” or “all” of the time, while an additional 58% said that they do so “most” of the time. In a more recent study of providers in the United States, 96% of respondents to the DocStyles 2016 survey reported that they screened patients for alcohol misuse [18].

While these findings are encouraging, practitioners’ self-reports may be subject to social desirability biases. Surveys reporting patients’ perspectives on SBI are scarce. One exception is a household survey study conducted in England in 2014, which described findings from patients who had visited their general practitioners within the previous 12 months and whose AUDIT scores suggested that they drank heavily. Of those, only 6.5% reported that they had received advice within this period concerning their drinking. The investigators also found that patients receiving advice were more likely to be male than female, but that patients’ age, “social grade” (a proxy for socioeconomic status), and race (white vs. non-white) were unrelated to their exposure to a brief intervention [19]. Investigators of another study of alcohol SBI conducted in five European jurisdictions found that only 2–10% of all patients were screened by their providers [20]. In contrast, findings from CDC’s 2017 Behavioral Risk Factor Surveillance System survey indicated that 81% of adults in its U.S. sample reported that they had been asked about their alcohol consumption by a health professional within the previous two years [3]. Summarizing the available literature, a recent review concluded that the proportion of hazardous or harmful drinkers who are identified as such through a screening process is probably very low [16].

Research to date has focused primarily on SBI prevalence in high-income countries. No study has investigated SBI prevalence in low- or middle-income countries, particularly among drinkers whose scores on a screening instrument indicate the need for intervention. Using samples of adult drinkers in Brazil, China, and South Africa, this study is the first to examine the prevalence of SBI in low- and middle-income countries.

Although we know very little about the prevalence of SBI outside of high-income countries, indicators are available about the prevalence of heavy episodic drinking (HED) and alcohol use disorder (AUD), the contribution of alcohol use to death and disability, and the extent of population-level access to health care, which may be important determinants. As shown in Table 1, the 2018 World Health Organization’s Global Status Report on Alcohol and Health indicates that past-30-day HED prevalence rates ranged from 18.3% in South Africa to 22.7% in China and were substantially higher among males compared to females [1]. Past-year AUD and alcohol dependence rates ranged from 4.2% in Brazil to 7.0% in South Africa and were also much higher among males [1]. The 2019 Global Burden of Disease study [21] found that alcohol use was the 6th leading risk factor for death and disability in Brazil, the 7th in South Africa, and the 8th in China. Based on these indicators, the need for SBI is apparent in these countries, particularly for males.

**Table 1:** Estimates of past-30-day prevalence of heavy episodic drinking and 12-month prevalence of alcohol use disorders and dependence (15 + years), by sex^1^

**Country**	**Heavy Episodic Drinking**			**Alcohol Use Disorder and Dependence**		
	**Male (%)**	**Female (%)**	**Both** **sexes (%)**	**Male (%)**	**Female (%)**	**Both** **sexes (%)**
Brazil	32.6	6.9	19.4	6.9	1.6	4.2
China	36.3	8.6	22.7	8.4	0.2	4.4
South Africa	30.6	6.5	18.3	12.4	1.8	7.0

^1^Source: World Health Organization. Global Status Report on Alcohol and Health. World Health Organization, Geneva; 2018

The World Health Organization’s Global Health Observatory provides estimates of the number of both medical doctors and nursing/midwifery personnel per 10,000 population [22]. The latest estimates suggest that the rates of these health care personnel, respectively, are 23.1 and 74.0 for Brazil, 19.8 and 26.6 for China, and 7.9 and 13.1 for South Africa. Additionally, the 2019 Universal Health Coverage Effective Coverage Index developed as part of the Global Burden of Disease study [23] indicates that China had a rating of 70, compared to 65 for Brazil and 60 for South Africa. While Brazil and China have universal health care systems, South Africa has a private system that primarily serves the affluent and a public health care system that serves the majority of the population [24]. In all three countries, the quality of health care in rural areas is poor relative to urban areas [24]. These indicators of population-level access to health care suggest that SBI may be more likely to occur in Brazil and China relative to South Africa, though the prevalence of AUD and alcohol dependence was higher in South Africa. The World Health Organization’s 2016 assessment of alcohol treatment services in 194 member countries also indicated that most of the improvement in the implementation of SBI in primary health care settings since 2010 was limited to upper-middle-income and high-income countries [1].

## Methods

### Samples

This study used data from surveys of household-based samples of adults conducted in 2018 as part of the Global Smart Drinking Goals (GSDG) evaluation [25]. This study was conducted in accordance with the Declaration of Helsinki and was reviewed and approved by the Institutional Review Board of the Pacific Institute for Research and Evaluation (FWA00003078). Respondents were told that their participation was voluntary and that their responses would be confidential. Only those who provided informed consent were interviewed.

Cities in the sample included Brasilia (subdistricts Ceilândia, Plano Piloto, Taguatinga) and Planaltina, Brazil; Jiangshan and Lanxi, China; and the Alexandra and Tembisa townships of Johannesburg, South Africa. A summary of the methods, sample sizes, and response rates is in Table 2. In each city, a multi-stage random sampling design was used along with quota sampling in some sites to ensure adequate sample sizes and statistical power. Survey weights were calculated using the ratio of census-based age and gender distributions of each city’s population relative to that of the survey sample to adjust for under- or over-representation of age and gender groups.

**Table 2:** Summary of survey methods, response rates, and sample sizes

**Country/City**	**Sample size**	**Response rate**	**Survey year and method**
Brazil	3,554	54.4%	April-May, 2018. Multi-stage random sample of census tracts and households with replacement, and one adult in each household. Quota sampling to achieve target sample size. In-person computer-assisted interviews with eligible adults.
Brasilia^1^	2,046	52.9%	
Planaltina	1,508	56.5%	
China	3,000	56.5%	May-June, 2018. Multi-stage random sample of village committees and households with replacement, and one adult in each household. Quota sampling to achieve target sample size. In-person computer-assisted interviews with eligible adults.
Jiangshan	1,500	47.4%	
Lanxi	1,500	69.9%	
South Africa	3,190	94.5%	Nov. 2018. Multi-stage random sample of small areas and households with replacement, and one adult in each household. Quota sampling to achieve target sample size based on age, gender, household type, and employment status in each area. In-person computer-assisted interviews with eligible adults.
Alexandra	1,484	92.4%	
Tembisa	1,706	96.5%	

^1^The subdistricts within Brasilia are Ceilândia, Plano Piloto, and Taguatinga

## Survey measures

### Past-year alcohol use

Respondents were asked whether they drank at least one alcoholic beverage (e.g., bottle of beer, glass of wine, shot of liquor or mixed drink) in the past 12 months. This study focused on respondents who answered “yes” to this question and answered subsequent questions about their alcohol use.

## Alcohol use disorder (AUD) risk

The four-item CAGE alcoholism screening instrument was used to assess AUD risk [26]: “At any time in the past 12 months…(a) have you felt that you should cut down on your drinking? (b) have people annoyed you by criticizing your drinking? (c) have you felt bad or guilty about your drinking? and (d) have you had a drink first thing in the morning to steady your nerves or to get rid of a hangover?” (0 = No, 1 = Yes). A summed score was computed for each respondent, and those with a score of 1 or higher were classified as being at risk for AUD as this threshold is more sensitive than the 2 + symptoms threshold for identifying hazardous drinkers who may be at risk for AUD [27, 28]. However, we also considered the 2 + symptoms threshold that is often used in research and clinical practice [29].

## Screening and brief intervention

Respondents were asked, “Have you talked about your health with a doctor, nurse, or other health care worker in the past 12 months?” Respondents who answered “yes” were then asked, “During the past 12 months, did any doctor, nurse, or other health care worker ask you about how much alcohol you drink, or have you fill out a form about this?” Past-year drinkers were classified as being screened for AUD risk if they responded “yes” to this question. These respondents were then asked, “During the past 12 months, did any doctor, nurse, or other health care worker advise you to reduce or stop drinking alcohol for some reason other than because you were starting a new medication or were pregnant?” Past-year drinkers were classified as receiving a brief intervention if they responded “yes” to this question.

## Sociodemographic characteristics

*Age, sex, and marital status.* Respondents in all cities were asked to report their age in years, sex (0 = female, 1 = male), and marital status (0 = not married, 1 = married).

*Ethnic/racial background*. Respondents in Brazil cities were asked, “What is your color or race?” (“White,” “Black,” “Asian,” “Brown,” “Indigenous,” and “Other”). Because only a small number of respondents classified themselves as “Indigenous,” they were recoded as “Other.” These variables were dummy coded with White as the referent group. Respondents in South Africa cities were asked, “What is your family’s native language?” (“Zulu,” “Sotho,” “Tsonga,” “Xosa,” “Afrikaans,” “English,” and “Other”). These variables were dummy coded with Zulu as the referent group.

*Education level*. Respondents in all three countries were asked, “What is your highest level of education?” The Brazil survey included nine possible response options (0 = Illiterate to 8 = Specialization/Master’s degree or above). The China survey had eight possible response options (1 = no formal education to 7 = university education and above). The South Africa survey had 16 possible response options (1 = No formal education to 16 = Post university education).

*Perceived wealth*. Respondents in the three countries were asked, “Compared with other families in [country], how rich or poor do you consider your family to be?” with seven response options (1 = poor to 7 = rich).

*Subjective health*. Respondents in all three countries were asked, “Considering the past 30 days, how satisfied are you with your overall health?” with five response options (1 = Very dissatisfied to 5 = Very satisfied).

### Data analysis

Descriptive analyses were conducted separately for each country to examine characteristics of past-year drinkers and to compare drinkers who did and did not report any CAGE symptoms with respect to whether they had talked to a health care professional in the past year, received a screening for AUD risk, and received advice about their drinking. In the subgroup of drinkers who had talked to a health care professional, we compared those with and without any CAGE symptoms on the survey with respect to whether they received screening for AUD risk and were advised about their drinking. We also compared percentages of drinkers across the three samples who received SBI.

Logistic regression analyses were conducted separately for drinkers in each country to determine whether having any CAGE symptoms was associated with receiving SBI in the past year when adjusting for demographic characteristics, subjective health, and perceived wealth. We conducted sensitivity analyses using the CAGE ≥ 2 symptoms threshold to determine whether this indicator risk was similarly associated with SBI. Analyses were conducted with HLM version 8.0 software to account for clustering of observations within each city and also within each country for between-country comparisons [30]. Sample weights were applied in all analyses.

## Results

### Sample characteristics

*Brazil.* Among drinkers in the Brazil sample, 63.8% reported talking to a health care provider in the past year (Table 3). A significantly lower percentage of drinkers with at least one CAGE (57.6%) symptom did so compared to those with no CAGE symptoms (70.8%). Of the total sample, 15.8% reported alcohol use screening in the past year, and 3.8% reported getting advice to stop or reduce their drinking. The percentage of drinkers with at least one CAGE symptom who received a brief intervention (5.6%) was significantly higher than the percentage of drinkers with no CAGE symptoms (1.8%). Relative to those with no CAGE symptoms, drinkers with at least one CAGE symptom were younger, more likely to be male or Black, and less likely to be White. These groups were similar on marital status. Relative to drinkers with no CAGE symptoms, those with at least one symptom also had lower levels of education, perceived wealth, and subjective health.

**Table 3:** Sample characteristics, mean (standard deviation) or percent^1^

**Variable**	**Brazil**			**China**			**South Africa**		
	**Total** **(N = 1,638)**	**CAGE = 0 (n = 853)**	**CAGE ≥ 1 (n = 785)**	**Total** **(N = 1,170)**	**CAGE = 0 (n = 818)**	**CAGE ≥ 1 (n = 352)**	**Total** **(N = 1,294)**	**CAGE = 0 (n = 956)**	**CAGE ≥ 1 (n = 338)**
Talked to health care provider in past year (%)	63.9	70.8	57.6**	25.3	24.6	27.0	35.2	35.6	35.1
Alcohol use screening in past year (%)	15.9	17.7	14.3	9.4	8.2	12.6**	5.6	2.4	6.7**
Brief intervention in past year (%)	3.8	1.8	5.6**	5.5	4.4	8.2**	3.6	0.6	4.6**
Age	35.6 (13.0)	37.4 (13.7)	34.0 (12.1)**	47.2 (16.4)	47.9 (16.3)	45.7 (16.6)	32.7 (10.6)	33.7 (12.4)	32.3 (9.9)*
Male (%)	56.4	49.2	63.0**	65.4	61.9	74.1**	70.0	58.5	74.1**
White (%)	29.4	34.7	24.4**	---	---	---	---	---	---
Black / Zulu (%)^2^	14.8	12.4	17.1*	---	---	---	31.3	32.0	31.1
Brown / Sotho (%)^2^	49.0	46.9	50.8	---	---	---	33.4	38.4	31.6*
Asian / Tsonga (%)^2^	3.3	2.9	3.6	---	---	---	10.7	9.7	11.1
Xosa (%)	---	---	---	---	---	---	8.0	6.8	8.4
Other (%)	3.5	3.1	4.0	---	---	---	16.6	13.2	17.8
Married (%)	54.9	56.4	53.5	80.4	81.6	77.4	20.3	24.0	19.0*
Education	4.0 (2.2)	4.5 (2.2)	3.6 (2.1)**	4.5 (1.9)	4.4 (1.1)	4.6 (1.3)	12.4 (2.5)	12.2 (2.9)	12.5 (2.4)
Perceived wealth	3.7 (1.3)	3.8 (1.2)	3.6 (1.3)*	3.9 (1.0)	3.9 (1.0)	3.9 (1.1)	3.0 (1.3)	3.0 (1.3)	3.0 (1.3)
Subjective health	3.9 (0.8)	3.9 (0.8)	3.8 (0.8)*	3.7 (0.7)	3.7 (0.7)	3.7 (0.8)	3.8 (0.9)	3.9 (0.9)	3.8 (1.0)

^1^Percentages and means are weighted, while sample (N) and subsample (n) sizes are unweighted

^2^The first ethnic/race group (e.g., Black) is for the Brazil sample, and the second native language group (e.g., Zulu) is for the South African sample

*p < .05, **p < .01; Statistical tests compared percentages or means among drinkers with and without any CAGE symptoms within each country

*China.* Among drinkers in the China sample, 25.3% reported talking to a health care provider in the past year, while 9.4% reported alcohol use screening and 5.5% reported receiving a brief intervention. The percentage of drinkers with any CAGE symptoms who reported alcohol use screening (12.6%) was significantly higher than the percentage of drinkers without any CAGE symptoms who received screening (8.2%), and a similar pattern was observed with respect to receiving a brief intervention (8.2% vs. 4.4%). Drinkers with any CAGE symptoms were more likely to be male than those without CAGE symptoms. There were no other significant differences in sociodemographic characteristics between the two groups.

*South Africa*. Among drinkers in the South Africa sample, 35.2% reported talking to a health care provider in the past year, while 5.6% reported alcohol use screening and 3.6% reported getting a brief intervention. Higher percentages of drinkers with any CAGE symptoms reported receiving an alcohol use screening (6.7%) and a brief intervention (4.6%) compared to drinkers without any CAGE symptoms (2.4% and 0.6%, respectively). Drinkers with at least one CAGE symptom were younger and more likely to be male or Sotho, and less likely to be married, than drinkers without any symptoms. These groups were similar with respect to other ethnicities and levels of education, perceived wealth, and subjective health.

## SBI among drinkers who talked to a health care professional

*Brazil.* Among drinkers in the Brazil sample who talked to a health care provider in the past year, 24.9% received an alcohol use screening, while 5.9% received a brief intervention (Table 4). There was no difference in the percentages of drinkers with and without any CAGE symptom who reported an alcohol use screening in the past year. However, the percentage of drinkers who received a brief intervention was significantly higher among those with CAGE symptoms (9.8%) than those without symptoms (2.5%).

**Table 4:** Screening and brief intervention among drinkers who talked to a health care provider in the past year, percent^1^

**Variable**	**Brazil**			**China**			**South Africa**		
	**Total** **(n = 1,046)**	**CAGE = 0 (n = 555)**	**CAGE ≥ 1 (n = 491)**	**Total** **(n = 308)**	**CAGE = 0 (n = 214)**	**CAGE ≥ 1 (n = 94)**	**Total** **(n = 457)**	**CAGE = 0 (n = 121)**	**CAGE ≥ 1 (n = 336)**
Alcohol use screening in past year (%)	24.9	25.0	24.8	38.0	33.5	48.3*	15.7	6.6	19.0**
Brief intervention in past year (%)	5.9	2.5	9.8**	22.0	18.0	31.5*	10.2	1.7	13.2**

^1^Percentages are weighted, while subsample (n) sizes are unweighted

*p < .05, **p < .01; Statistical tests compared percentages among drinkers with and without any CAGE symptoms within each country

*China.* Among drinkers in the China sample who talked to a health care provider in the past year, 38.0% received alcohol use screening and 22.0% received a brief intervention. Higher percentages of drinkers with at least one CAGE symptom reported alcohol use screening (48.3%) and receiving a brief intervention (31.5%) than did drinkers without any symptoms (33.5% and 18.0%, respectively).

*South Africa.* Among drinkers in the South Africa sample who talked to a health care provider in the past year, 15.7% reported alcohol use screening and 10.2% reported receiving a brief intervention. Higher percentages of drinkers with at least one CAGE symptom reported alcohol use screening (19.0%) and receiving a brief intervention (13.2%) than did drinkers without any symptoms (6.6% and 1.7%, respectively).

## Comparisons of SBI prevalence across samples

Analyses indicated that the prevalence of alcohol use screening was significantly higher among all drinkers in the Brazil sample compared to those in China [χ^2^ (1) = 26.0, p < .01] and South Africa [χ^2^ (1) = 78.4, p < .01], while there was no difference in the prevalence of alcohol use screening among drinkers in China and South Africa. There were no significant differences between the three samples in the prevalence of receiving advice from a health care provider.

Among drinkers who had talked to a health care professional in the past year, the prevalence of alcohol use screening was significantly lower in South Africa compared with Brazil [χ^2^ (1) = 15.7, p < .01] and China [χ^2^ (1) = 47.5, p < .01]. The prevalence of alcohol use screening was significantly lower among drinkers in the Brazil sample compared to those in the China sample [χ^2^ (1) = 18.9, p < .01]. The prevalence of receiving advice from a health care provider was also significantly higher among drinkers in China than those in Brazil [χ^2^ (1) = 69.8, p < .01] or South Africa [χ^2^ (1) = 20.3, p < .01]. The prevalence of receiving a brief intervention was lower among drinkers in the Brazil sample compared to those in the South Africa sample [χ^2^ (1) = 8.5, p < .01].

## Predicting likelihood of SBI

*Brazil.* Logistic regression analyses adjusting for demographic characteristics (Table 5) indicated that among drinkers in the Brazilian sample, reporting CAGE symptoms on the survey was not associated with receiving alcohol use screening. Higher levels of education and perceived wealth were positively associated with alcohol use screening. However, drinkers with at least one CAGE symptom were more than three times as likely to receive a brief intervention as drinkers without any symptoms when adjusting for sociodemographic characteristics. Age was positively associated with receiving a brief intervention.

**Table 5:** Results of logistic regression analyses to assess predictors of screening and brief intervention, odds ratio (95% confidence interval)^1^

**Variable**	**Brazil**		**China**		**South Africa**	
	**Screening**	**Brief intervention**	**Screening**	**Brief intervention**	**Screening**	**Brief intervention**
Age	1.01 (0.99, 1.02)	1.02 (1.00, 1.04)*	1.02 (1.00, 1.04)*	1.01 (0.99, 1.03)	1.05 (1.03, 1.08)**	1.07 (1.04, 1.10)**
Male	0.96 (0.73, 1.27)	1.00 (0.58, 1.74)	1.47 (0.87, 2.45)	1.98 (0.96, 4.11)	1.22 (0.69, 2.18)	1.05 (0.51, 2.17)
Black^2^	0.98 (0.62, 1.53)	0.93 (0.35, 2.54)	---	---	---	---
Brown / Sotho^2,3^	1.16 (0.84, 1.59)	1.57 (0.80, 3.09)	---	---	0.54 (0.26, 1.09)	0.31 (0.11, 0.86)**
Asian / Tsonga^2,3^	0.96 (0.41, 2.23)	2.16 (0.56, 8.29)	---	---	1.54 (0.72, 3.29)	1.98 (0.82, 4.79)
Xosa^2^	---	---	---	---	1.41 (0.60, 3.31)	1.67 (0.61, 4.58)
Other^2^	0.58 (0.22, 1.54)	1.52 (0.40, 5.76)			0.96 (0.47, 1.98)	0.84 (0.34, 2.11)
Married	1.29 (0.97, 1.72)	1.69 (0.95, 3.00)	1.19 (0.65, 2.20)	1.94 (0.81, 4.61)	1.00 (0.54, 1.89)	1.19 (0.56, 2.51)
Education	1.12 (1.05, 1.19)**	0.94 (0.84, 1.07)	0.87 (0.71, 1.06)	0.84 (0.66, 1.08)	1.05 (0.95, 1.15)	1.06 (0.94, 1.19)
Perceived wealth	1.17 (1.04, 1.30)**	1.05 (0.85, 1.30)	0.98 (0.79, 1.21)	0.90 (0.70, 1.17)	0.97 (0.80, 1.18)	1.09 (0.86, 1.38)
Subjective health	0.89 (0.76, 1.05)	0.79 (0.60, 1.04)	0.90 (0.67, 1.20)	0.72 (0.51, 1.01)	0.78 (0.62, 0.98)*	0.78 (0.59, 1.04)
CAGE ≥ 1	0.92 (0.69, 1.21)	3.39 (1.81, 6.36)**	1.98 (1.22, 3.20)**	2.19 (1.22, 3.93)**	3.12 (1.42, 6.89)**	11.2 (2.24, 55.7)**

^1^Regression analyses included all past-year drinkers

^2^White is the referent group for the Brazil sample and Zulu is the referent group for the South African sample

^3^The first ethnic/race group (e.g., Brown) is for the Brazil sample, and the second native language group (e.g., Sotho) is for the South African sample

*p < .05, **p < .01

*China.* After adjusting for sociodemographic characteristics, drinkers with at least one CAGE symptom were about twice as likely to report alcohol use screening or receiving a brief intervention as drinkers without any CAGE symptoms. Age was also positively associated with alcohol use screening.

*South Africa.* Drinkers in the South Africa sample with at least one CAGE symptom were more than three times as likely to report alcohol use screening and about 11 times as likely to receive a brief intervention than drinkers without any symptoms when adjusting for sociodemographic characteristics. Age was positively related to both alcohol use screening and brief intervention; Sotho ethnicity was inversely associated with receiving a brief intervention. A higher level of positive subjective health was inversely related to alcohol use screening.

## Sensitivity analyses

Supplemental Table 1 summarizes the results of sensitivity analyses with CAGE ≥ 2 symptoms as the threshold. These analyses indicate associations of a positive CAGE threshold with SBI for drinkers in sampled Brazilian and Chinese cities, but weaker, though still significant associations with SBI among drinkers in the sampled South African townships.

## Discussion

This study was the first to investigate the prevalence of alcohol SBI in middle-income countries, focusing on adult drinkers in cities in Brazil, China, and South Africa. This is also one of very few studies to assess SBI from the perspective of health care recipients, rather than professionals. Similar to other studies in high-income countries [16], our findings indicate very low prevalence rates of SBI among drinkers at risk for AUD, regardless of whether they reported talking to a health care professional in the previous year. Although the higher prevalence rates of SBI among drinkers who did report talking to a health care professional point to the potential importance of primary care settings, the overall low rates of screening and intervention, even among those who screen positive, indicate that efforts are needed to encourage health care workers to consistently implement SBI. Providing additional training and incentivizing health care workers to routinely ask about alcohol use and intervene when indicated may help increase the prevalence of SBI in order to achieve population-level reductions in hazardous drinking. Increasing availability of appropriate digital technology (e.g., e-SBI administered via computer or mobile phone) could also help to increase SBI as a routine part of primary health care [32].

Our expectation that SBI prevalence would be higher in Brazil and China was partially supported, in that the alcohol use screening prevalence rates were higher among drinkers in those samples than in the South Africa sample overall. However, brief intervention prevalence rates were not significantly different among drinkers in the three samples overall, and were lower among drinkers in Brazil who talked to a health care professional compared to those in the South Africa sample. Prevalence rates for SBI were significantly higher among drinkers who had talked to a health care professional in the China sample compared to those in Brazil and South Africa. Although these findings suggest that greater availability of primary care providers may help to increase screening of drinkers for AUD risk, greater attention is needed to promote SBI and provide appropriate training for health care professionals.

Findings from this study add to the scant research available on the association between the likelihood that patients will receive SBI and their sociodemographic characteristics. In the only prior study we could find that addressed this issue, receipt of SBI was linked to sex, but not to race, age, or socioeconomic status [19]. In contrast, this study found a positive association between age and the prevalence of either screening (in China), brief intervention (Brazil), or both (South Africa). Further, positive associations were found in Brazil between screening and both education and perceived wealth, and in South Africa between screening and subjective health. These findings suggest the need for further studies of potential biases by health providers that may adversely affect delivery of SBI to the broader population of drinkers.

Based on the CAGE ≥ 1 symptom threshold, AUD risk was associated with greater odds of receiving a brief intervention among drinkers in the Brazil sample, and with greater odds of both screening and brief intervention in the China and South Africa samples when adjusting for sociodemographic characteristics. Similar results were observed with the CAGE ≥ 2 symptoms threshold for AUD risk. These findings suggest that drinkers at risk for AUD in Brazil, China, and South Africa are more likely to receive advice from a health care professional about their drinking than drinkers not at risk for AUD, but it is not clear how much of this is the result of primary preventative care versus treatment for alcohol-related health problems. Further research is needed to address this question.

Limitations of this study include its use of adult samples from only two cities within each of three countries that may not be representative of all adults in each country, and survey response rates in Brazil and China cities were less than optimal. Some questions (e.g., race/ethnicity) were asked differently in each country to accommodate cultural differences, which may affect cross-country comparisons. While the use of a threshold of 1 for the CAGE has precedents in the research literature [27, 28], some studies have applied a higher cutoff [29], and others have relied on the AUDIT for AUD screening [31]. Findings of studies may vary by differences in the screening tools and cutoff scores employed by investigators. Additionally, this study did not assess other cultural and structural factors that may be important determinants of SBI, including SBI implementation as part of a national health policy to address alcohol problems, SBI training in medical and nursing schools, and SBI implementation in hospitals and primary care clinics. These factors should be considered in future studies on SBI.

In summary, this study is among very few that have described SBI prevalence in middle-income countries or from the perspective of health care recipients. Our findings indicate very low prevalence rates of SBI among drinkers at risk for AUD, even if they had talked to a health care professional in the previous year. More efforts to promote the consistent use of SBI in primary care settings are clearly warranted.

## Electronic supplementary material

Below is the link to the electronic supplementary material.

Supplementary Material 1

## Data Availability

Survey data used for this study are available upon request from Dr. Paschall, and are stored in the Global Smart Drinking Goals Data Library (https://gsdgdatalibrary.org).
