# Supplementary material for: Screening and brief intervention for alcohol use disorder risk in three middle-income countries
Source: BMC Public Health. 2022 Oct 26;22:1967. doi: 10.1186/s12889-022-14358-4 (PMC9609268; doi:10.1186/s12889-022-14358-4)
Supplement: Supplementary file 1 — Supplementary Material 1 [file 12889_2022_14358_MOESM1_ESM.docx]

**Supplemental Table 1** Summary results of logistic regression sensitivity analyses, odds ratio (95% confidence interval)^1^

| **Variable** | **Brazil** | | **China** | | **South Africa** | |
| --- | --- | --- | --- | --- | --- | --- |
|  | **Screening** | **Brief intervention** | **Screening** | **Brief intervention** | **Screening** | **Brief intervention** |
| CAGE ≥ 2 | 1.03 (0.76, 1.39) | 3.53 (2.01, 6.20)** | 2.09 (1.16, 3.79)** | 2.18 (1.07, 4.48)** | 1.83 (1.08, 3.11)** | 2.64 (1.31, 5.32)** |

^1^Regression analyses included all past-year drinkers, and results are adjusted for participants’ sociodemographic characteristics.

*p < .05, **p < .01
